# Development of a capillary-modified naked-eye visual loop-mediated isothermal amplification method for the rapid detection of mpox virus and chikungunya virus

**DOI:** 10.3389/fmicb.2025.1615132

**Published:** 2025-08-05

**Authors:** Junwen Luan, Shuai Song, Chen Cheng, Daoqun Li, Liyuan Zhu, Huixiang Cheng, Leiliang Zhang

**Affiliations:** ^1^Department of Clinical Laboratory Medicine, The First Affiliated Hospital of Shandong First Medical University and Shandong Provincial Qianfoshan Hospital, Jinan, China; ^2^Department of Pathogen Biology, School of Clinical and Basic Medical Sciences, Shandong First Medical University and Shandong Academy of Medical Sciences, Jinan, China

**Keywords:** LAMP, capillary, MPXV, CHIKV, diagnostic method

## Abstract

The global emergence of mpox virus (MPXV) and chikungunya virus (CHIKV) has intensified the demand for advanced diagnostic methods. Rapid, sensitive, cost-effective diagnostic methods are crucial for preventing cross-border transmission and early containment of community spread. In this study, we developed a capillary modified Loop-mediated isothermal amplification (LAMP) assay for the identification of MPXV and CHIKV. This system employs capillaries as reaction vessels, offering advantages such as reduced reagent consumption and simplified operation. The capillary-driven liquid handling system also significantly reduces the frequency of lid openings during reagent transfer compared to standard LAMP protocols. This minimizes the risks of aerosol contamination and the associated false-positive outcomes that are inherent to conventional methods. Additionally, direct visual interpretation of the results without specialized instrumentation is achieved through integration of a leuco-hydroxynaphthol blue (LHNB) dye. This novel detection method targets the F13 gene of MPXV, the nsP1 gene of CHIKV, live vaccinia virus (VACV) and CHIKV viruses. Analytical sensitivity reached 10 copies/μL for MPXV F13 and 6 copies/μL for CHIKV nsP1. Because of the high level of laboratory biosafety required for MPXV culture, VACV was selected as a safe surrogate model for detection, where the E9L gene was selected to target all *Orthopoxvirus* (OPXV). The detection limits of infectious units for intracellular and extracellular viruses of VACV are 0.64 plaque-forming units (PFU) and 8 PFU, respectively. For CHIKV infection, the detection limits of infectious units for intracellular and extracellular viruses are 0.3 PFU and 0.068 PFU, respectively. The capillary modified LAMP assay achieves higher sensitivity to current gold-standard qPCR assays, while offering several advantages, including rapid turnaround time (results obtained within 30 min), minimal equipment requirements (single heating module), cost-effectiveness, visual readout compatibility, and no requirement for specialized personnel. This study confirmed the capacity of this improved LAMP colorimetric detection method. The system addresses critical gaps in resource-limited scenarios, offering a deployable solution for border quarantine stations and primary healthcare services–key nodes for intercepting cross-border transmission and mitigating localized outbreaks through timely case identification.

## 1 Introduction

Mpox, caused by the mpox virus (MPXV), is classified as a highly infectious zoonotic viral disease within the *Orthopoxvirus* genus of the *Poxviridae* family (Li et al., 2023; Moss, 2024). Its genome comprises a double-stranded DNA molecule spanning approximately 200 kb and encoding around 190 genes. Characteristic clinical manifestations, including painful rashs [often begins on the face and spreads over the body, fever, muscle aches and lymphadenopathy, which overlap with smallpox symptoms, potentially leading to diagnostic misinterpretation (Moss, 2024)]. The first human case of mpox was reported in the Democratic Republic of Congo in 1970 (Breman et al., 1980). Since then, it frequently occurs in Central and West African countries, especially in the Democratic Republic of Congo, where it was considered endemic. Notably, in 2022 a new outbreak of mpox occurred in countries beyond Africa regions. Over the past years, the World Health Organization (WHO) had twice declared MPXV a Public Health Emergency of International Concern (PHEIC) (Dutta et al., 2025).

Chikungunya fever (CHIKF), caused by the mosquito-borne chikungunya virus (CHIKV), is characterized by acute febrile illness associated with severe, often debilitating polyarthralgias (Kril et al., 2021). Typical symptoms include abrupt onset of fever and severe joint pain. It is often clinically indistinguishable from dengue or Zika virus infections. The first case of CHIKF was found in the United Republic of Tanzania in 1952, and other cases were recorded in countries in Africa and Asia since then (Nsoesie et al., 2016). However, no major outbreak happened up until 2004 when a large epidemic started on the coast of Kenya, which lasted for 5 years and spread across India and parts of Southeast Asia. As of now, CHIKV has been documented in over 110 countries, demonstrating high transmissibility and establishing itself as a globally emerging public health priority (Cai et al., 2022).

Current gold standards for both MPXV and CHIKV diagnosis rely on quantitative polymerase chain reaction (qPCR) assay, which offers outstanding sensitivity and specificity (Liu et al., 2024; Shahrtash et al., 2025). However, the substantial operational costs of qPCR simply render it impractical for large-scale screening. Not to mention the method of qPCR requires specialized laboratory instrument and skilled personnel creating a bottleneck when it comes to implementation in resource-constrained settings, namely primary healthcare services and border control points, which are the frontlines of disease prevention and control. These limitations impede rapid pathogen detection during outbreaks, potentially exacerbating epidemic spread due to delayed diagnosis. Consequently, there is an urgent need for a simplified diagnostic method that combines rapidity, cost-effectiveness, and minimal technical requirements to enhance outbreak preparedness in frontline healthcare systems.

Loop-mediated isothermal amplification (LAMP) emerges as a viable solution to this problem (Notomi et al., 2000). It is an enzyme-driven nucleic acid amplification methodology, also characterized by high sensitivity, yet offering operational simplicity, higher amplification speed and cost reduction compared to qPCR while maintaining diagnostic concordance (Kang et al., 2022). The LAMP technique relies on four to six specially designed primers that can recognize specific regions on the target sequence and a DNA polymerase with strand displacement activity (Soroka et al., 2021). Under the isothermal conditions, it can rapidly amplify the target sequence with high specificity. This method can amplify up to 10^9^ copies of target DNA within an hour at a constant temperature of approximately 65°C (Yu et al., 2021). Due to the absence of temperature cycling, the reaction conditions are easy to control, and the equipment required is simple. Moreover, it is as sensitive and specific as traditional PCR methods. As a result, LAMP has become a major direction for future nucleic acid detection and offers distinct advantages compared to qPCR and serological detection methods, making it particularly suitable for decentralized MPXV/CHIKV detection.

**Figure 1:**
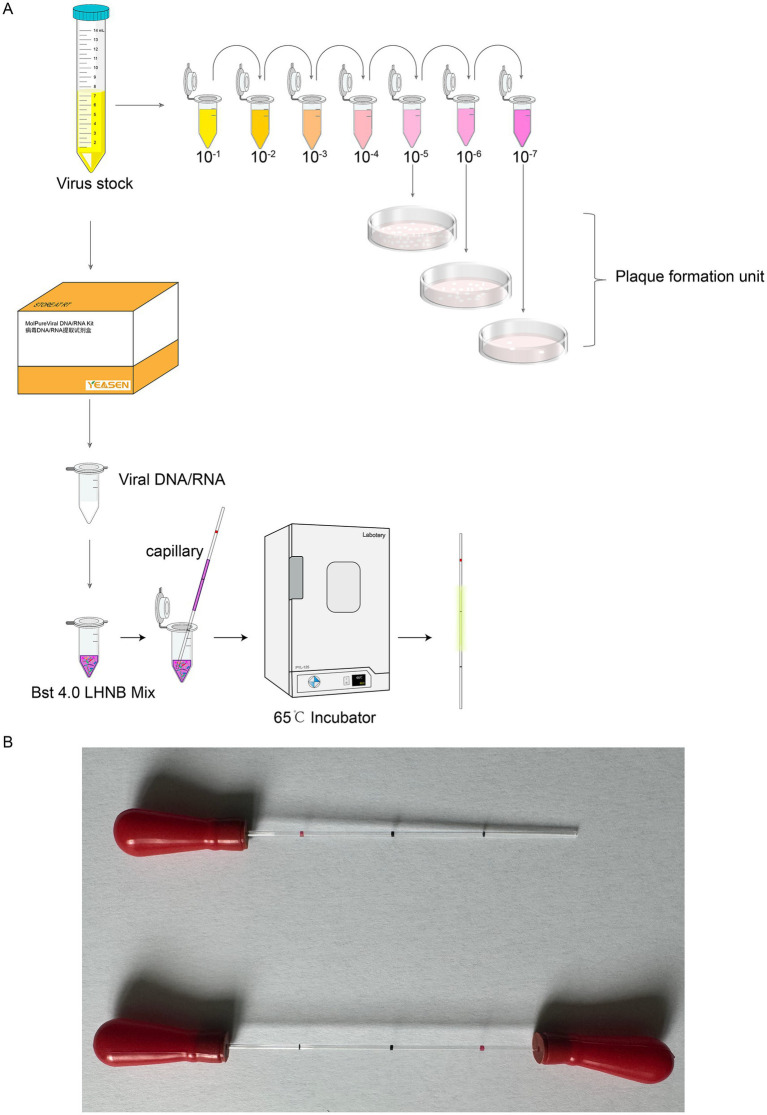
Experimental process schematic of capillary-modified naked-eye visual LAMP method for detection of viral infectivity. **(A)** Outline the experimental design. **(B)** The capillaries with rubber tips in the end.

To bridge the diagnostic accessibility gap, we take it a step further, engineered a novel detection system based on capillary-modified LAMP, as depicted in Figure 1A for rapid screening of MPXV and CHIKV. The whole reaction is done in a capillary (Figure 1B) and less reagents is used. Using of capillaries resulted in easier distinction of color changes and greatly simplified the operation. Subsequently, we developed and validated our detection methods using plasmids and live viruses. Results can be visualized within 30 min after sampling, and the sensitivity of our assay was comparable to that of qPCR.

## 2 Materials and methods

### 2.1 Primer design

According to the literatures (Van Vliet et al., 2009; Zhao et al., 2023), we selected the highly conserved and specific E9L as the target gene indicator for detecting vaccinia virus (VACV). Ssequences of the E9L gene from the VACV WR strain (GenBank: AY243312.1), mpox virus (GenBank: ON563414.3), Camelpox virus (GenBank: AF438165.1), and Horsepox virus (GenBank: DQ792504.1) were also downloaded from NCBI. These sequences were subjected to multiple sequence alignment in Clustal Omega. The conserved region (2,392–2,779 bp) was selected, and sequences from this region were imported into the PrimerExplorer V5 software,^1^ resulting in the identification of the best specific primers for VACV.

Based on existing research (Wesselmann et al., 2024), we chose the highly conserved and specific nsP1 as the target gene indicator for detecting CHIKV. Sequences of the nsP1 gene from the CHIKV 181/25 strain (GenBank: MW473668.1), Western equine encephalitis virus (NC_075015.1), Eastern equine encephalitis virus (NC_003899.1), Venezuelan equine encephalitis virus (NC_075022.1), Ross River virus (NC_075016.1), and Mayaro virus (NC_003417.1) were downloaded from NCBI. These sequences were imported into Clustal Omega for multiple sequence alignment. The region exhibiting the greatest differences (1,350–1,605 bp) was selected, and sequences from this region were imported into the LAMP primer design software PrimerExplorer V5, leading to the identification of the most specific primers for CHIKV.

Additionally, since MPXV F13 is the target for the antiviral drug tecovirimat, we selected F13 as the target gene for detecting MPXV. The position of F13 gene fragment is from position 39,605 bp to 39,830 bp. The LAMP primers used in this study were designed using the LAMP PrimerExplorer V5 software. The primers included the forward outer primer F3, backward outer primer B3, forward inner primer FIP, backward inner primer BIP, loop forward primer LoopF and loop backward Primer LoopB (Table 1). The reference sequences for the target regions used in primer design were sourced from the NCBI GenBank database. All oligonucleotide are purified by PAGE in Beijing Qingke Biotechnology Co., Ltd., Jinan Oligonucleotide Synthesis Department.

**Table 1:** Primer sets used for the MPXV and CHIKV LAMP assay.

Primers^a^	Sequence (5′-3′) and modifications	Length^b^
MPXV F13-F3	GATGTTATGTAGGAAACGCG	20 nt
MPXV F13-B3	AACACTCCACCTATAGGATTC	21 nt
MPXV F13-FIP	GCCAGCGGAGGATAATCAGAATATATTTACTGGAGGATCTATACATACG	49 nt
MPXV F13-BIP	ATAGCGCAAAAAATTCATGGTTGAAATATGATACGCAGTGCTAAC	45 nt
CHIKV nsP1-F3	ATCCTTGCTACAGAAGTCA	19 nt
CHIKV nsP1-B3	AGCAGCAGGTTAGTGTT	17 nt
CHIKV nsP1-FIP	CATGGTGTTCGTGTTCCGTGGCTGAACCAGAGGATAG	37 nt
CHIKV nsP1-BIP	ACCTACTTCCCGTGGTCGTCTTCCATGTCCTTCCG	35 nt
CHIKV nsP1-LoopF	GCGTTCTGCCGTTAACCA	18 nt
CHIKV nsP1-LoopB	AAGTGGGCAAAGGAGTGC	18 nt
VACV E9L-F3	CCGAACCTCATCTCTGAA	18 nt
VACV E9L-B3	CTGTAATCTATAGGCAACGA	20 nt
VACV E9L-FIP	TGCATGGAATCATAGATGGCCGATAGAAGGAACCATTCC	39 nt
VACV E9L-BIP	ACTCATACGCTTCGGCTAAGACCGTAACATACCGTTAGAT	40 nt
VACV E9L-LoopF	CAGTTGAACTGGTAGCCTGT	20 nt
VACV E9L-LoopB	AGTTGCACATCCATAGGACG	20 nt

a F3, forward outer primer; FIP, forward inner primer; BIP, backward inner primer; B3, backward outer primer; LoopF, loop forward primer; LoopB, loop backward Primer.

b nt, nucleotide; mer, monomeric unit.

### 2.2 Plasmids and viruses

The nsP1 gene of CHIKV 181/25 (GenBank: MW473668.1) was cloned into pCMV-14 by Beijing Qingke Biotechnology Co., Ltd. The F13 gene of MPXV USA 2022MA001 (NCBI GenBank: ON563414.3) was cloned into pEGFP-C1 by Beijing Qingke Biotechnology Co., Ltd. Vaccinia virus (VACV) WR stain and CHIKV 181/25 strain (Levitt et al., 1986) are stored in our laboratory.

### 2.3 Cell culture

BSC-1 cells are a continuous cell line derived from the kidney tissue of the African green monkey (*Cercopithecus aethiops*) (Hopps et al., 1963),^2^ and are widely used in virology research. This cell line was employed in the plaque assays for VACV in this study. BSC-1 cells were cultured in RPMI 1640 medium supplemented with 10% fetal bovine serum (FBS) and 1% penicillin/streptomycin at 37°C in a 5% CO_2_ atmosphere. Vero cells (for CHIKV culture) (Higashi et al., 1967) or HeLa cells (for VACV culture) (Scherer et al., 1953) were seeded in DMEM medium supplemented with 10% FBS and 1% penicillin/streptomycin at 37°C in a 5% CO_2_ atmosphere.

### 2.4 Virus propagation

Cell growth was monitored to ensure reaching 80–90% confluency. At this stage, an appropriate amount of previously stored CHIKV or VACV viral stock was added to the culture. The infected cells were then incubated in a cell culture incubator until evident cytopathic effects (CPE) appeared, as observed under an inverted microscope. Typical manifestations of CPE included cell rounding, shrinkage, clustering into grape-like formations, followed by detachment and lysis of the monolayer, along with a color change in the culture medium to yellow. Supernatants and cells were harvested and stored at −80°C. This freeze–thaw cycle was repeated three times to ensure complete viral release from the cells. The final viruses were aliquoted, stored at −80°C, and labeled with the virus type, infection time, host cell line, and number of freeze–thaw cycles. Prior to each use, the virus stock was briefly subjected to ultrasonication in an ice-water bath to ensure uniform dispersion of viral particles.

### 2.5 Preparation of 25× LAMP primer mix

The concentrations of FIP/BIP primers were 25 μM, LoopF/B primers were 4–8 μM, and F3/B3 primers were 5 μM, respectively. ddH_2_0 was used as the primer dilution, and the primer system was made up to 0.8 μL with ddH_2_O.

### 2.6 Visual detection of LAMP amplification using leuco-hydroxynaphthol blue dye

The leuco-hydroxynaphthol blue (LHNB) dye in the reaction system binds to Mg^2+^, producing a red color before the reaction begins. As the LAMP reaction proceeds, the inorganic pyrophosphate (PPi) generated by positive samples binds to the Mg^2+^ in the system, resulting in a yellow-green color for the LHNB, while the negative control remains unchanged (Zaczek-Moczydłowska et al., 2020; An et al., 2025). The detailed introduction is shown in the product webpage.^3^

### 2.7 Reaction system configuration

The reaction system we used (HotStart Bst 4.0 LHNB Lyo Mix) contains reverse transcriptase activity, allowing the RT-LAMP reaction to be performed without the need for additional reverse transcriptase (Chander et al., 2014; Paik et al., 2023). A 25 μL standard CHIKV reaction system and an MPXV reaction system optimized to 10 μL were set up for further exploring the optimization of reaction reagents with capillary tubes. Standard CHIKV nsP1 plasmid and CHIKV RNA extracted from live CHIKV virus reaction system 20 μL: Bst4.0 LyoMixA 1 μL, Bst4.0 LyoMixB 7.5 μL, 25 × LAMP Primer Mix 0.8 μL, and template RNA 10 μL to be added followed, ddH_2_O up to a total volume of 20 μL. MPXV F13 plasmid and DNA extracted from live VACV reaction system was subjected to a series of tests and adjustments to improve its performance at 10 μL. Finally, the following configuration was found to obtain the best results, with the reaction system optimized to 10 μL: Bst4.0 LyoMixA 0.6 μL, Bst4.0 LyoMixB 4.5 μL, 25 × LAMP Primer Mix 0.4 μL, and template DNA 5 μL to be added followed, ddH_2_O up to a total volume of 10 μL. The components and their volumes are listed in Table 2.

**Table 2:** LAMP reaction system.

Component	Volume
HS-Bst4.0 LyoMixA	0.5 μL
HS-Bst4.0 LyoMixB	3.75 μL
FIP/BIP	0.12 μL
F3/B3	0.02 μL
LoopF/LoopB	0.04 μL
50xLHNB Dye	0.2 μL
Nucleic acid template	5 μL
ddH_2_O	0.19 μL
Total	10 μL

### 2.8 Kits used in this study

HotStart Bst 4.0 LHNB Lyo Mix (Cat No. A3824-43) and Bst 4.0 LS MasterMix (Cat No. A3824-01) were from Haigene, Harbin, China. The capillary reaction system employed either the HotStart Bst 4.0 LHNB Lyo Mix, which exhibits a color change from violet (negative) to green (positive), or the Bst 4.0 LS MasterMix, which shows a color change from red (negative) to yellow (positive). Viral DNA and RNA were isolated through MolPure^®^ Viral DNA/RNA Kit (Cat No. 19321ES50), which was from Yeasen, Shanghai, China. The plasmid was purified by GoldHi EndoFree Plasmid Midi Kit (Cat No. CW2581), which was from Cwbio, Beijing, China.

### 2.9 Capillary-modified LAMP reaction process

The capillary tube is a widely used disposable micro blood collection device for clinical purposes (registration certificate number: Sutaixiebei 20160157). This material is sourced from Kangjianhua, Jiangsu, China. The conventional method is to aspirate a solution from a pre-mixed enzymatic reaction system using a pipette and then aspirate the template DNA/RNA and add it to the 0.2 mL single PCR tubes (The catalog number: 401001, NEST, Wuxi, Jiangsu, China) for reaction. The modification of the method is to take a capillary tube to draw up the solution in the enzymatic reaction system that has been mixed in advance, and then aspirate the corresponding sample to be tested, and coat both ends of the capillary tube with mineral oil and apply a capillary glue tip to make it sealed. Place the capillary in a constant temperature incubator at 65°C for 30 min, and observe the color change of the capillary during the reaction.

### 2.10 Agarose gel electrophoresis analysis to determine performance of optimized LAMP amplification system

PCR and LAMP amplification capabilities were compared using 2% agarose gel electrophoresis analysis, where PCR targets the same genes as LAMP. The experiment was repeated 3 times to check reproducibility and specificity.

### 2.11 Plaque assay

BSC-1 cells were seeded into a 6-well plate and incubated until they reached the desired confluency. The virus stock was serially diluted in RPMI 1640 medium to final concentrations of 10^−1^, 10^−2^, 10^−3^, 10^−4^, 10^−5^, 10^−6^, and 10^−7^. The diluted virus solutions were then added to the wells containing the pre-seeded cells. The plates were incubated at 37°C in a CO₂ incubator for 2 h. After incubation, the viral inoculum was aspirated, and the wells were washed three times with phosphate-buffered saline (PBS). The 6-well plates were then returned to the CO₂ incubator for further incubation. Cells were allowed to incubate for an additional 2.5–3 days until clear plaque formation was observed.

## 3 Results

### 3.1 Preliminary experiment

**Figure 2:**
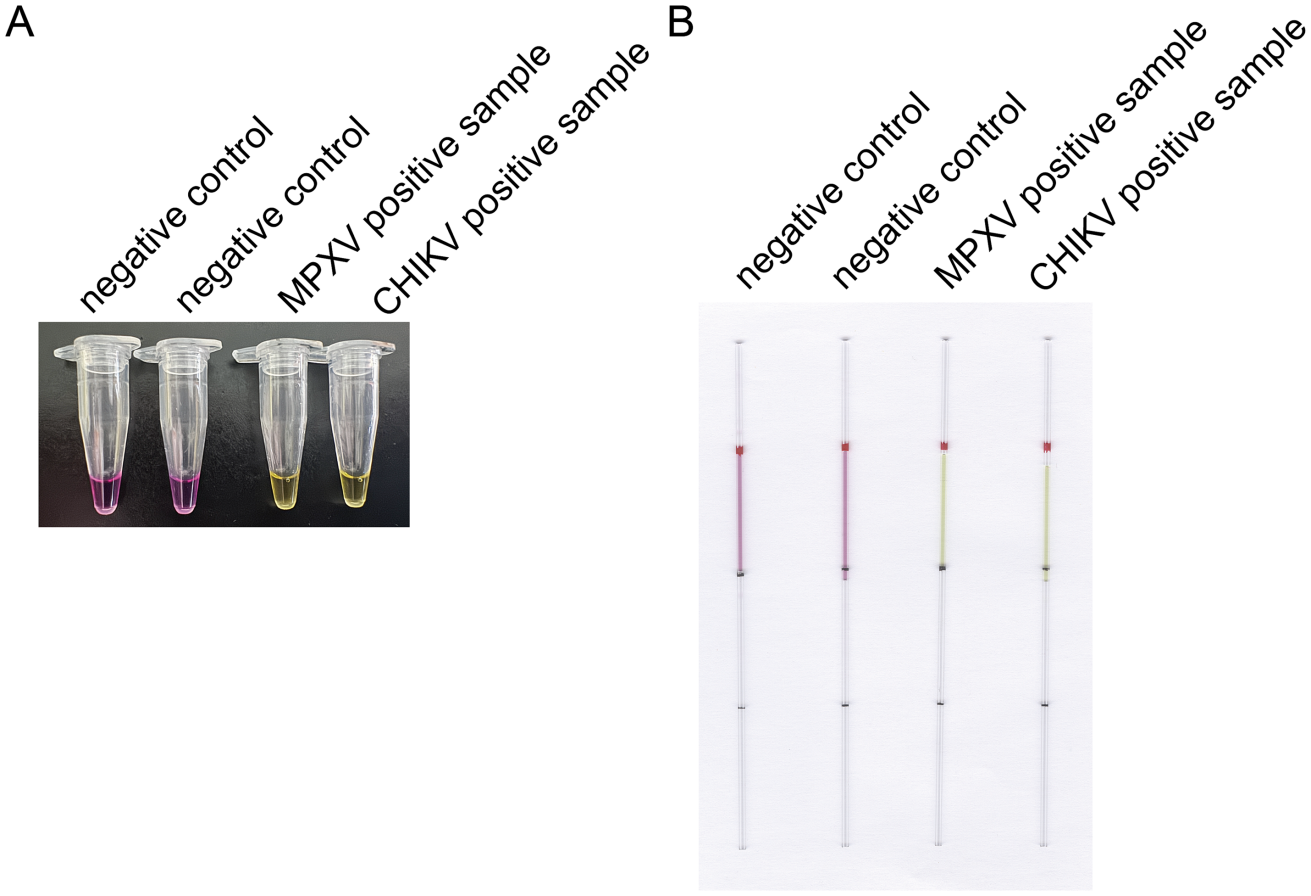
Preliminary experiment for the optimized LAMP detection. **(A)** Preliminary experiments using microcentrifuge tube for LAMP detection. **(B)** Preliminary experiments with a modified LAMP assay using capillaries. This capillary reaction system utilized the HotStart Bst 4.0 LHNB Lyo Mix that features a violet (negative) to green (positive) color change.

The capillaries are made of high borosilicate glass and have undergone silanization treatment, with a capacity accuracy of <±1%, which allows for a more intuitive visualization of the reaction color compared to conventional PCR tubes or microcentrifuge tubes. The capillaries may roll and potentially drop and break during handling, which can lead to additional complications such as aerosol contamination and sharps hazards. To mitigate these risks, we added rubber tips to the ends of the capillaries (Figure 1B). The capillary is designed to accurately aspirate 10 μL of liquid, featuring graduated markings along its length. The volume between each pair of markings corresponds to 10 μL (Figure 1B). A preliminary experiment was performed using microcentrifuge tube/capillary and the standard CHIKV reaction system, as well as the modified MPXV reaction system for loop-mediated isothermal amplification. The results consistently showed a color change in the microcentrifuge tube/capillary from violet to green, and the negative control group did not change (Figures 2A,B).

### 3.2 Optimization of the LAMP reaction system and specificity validation

**Figure 3:**
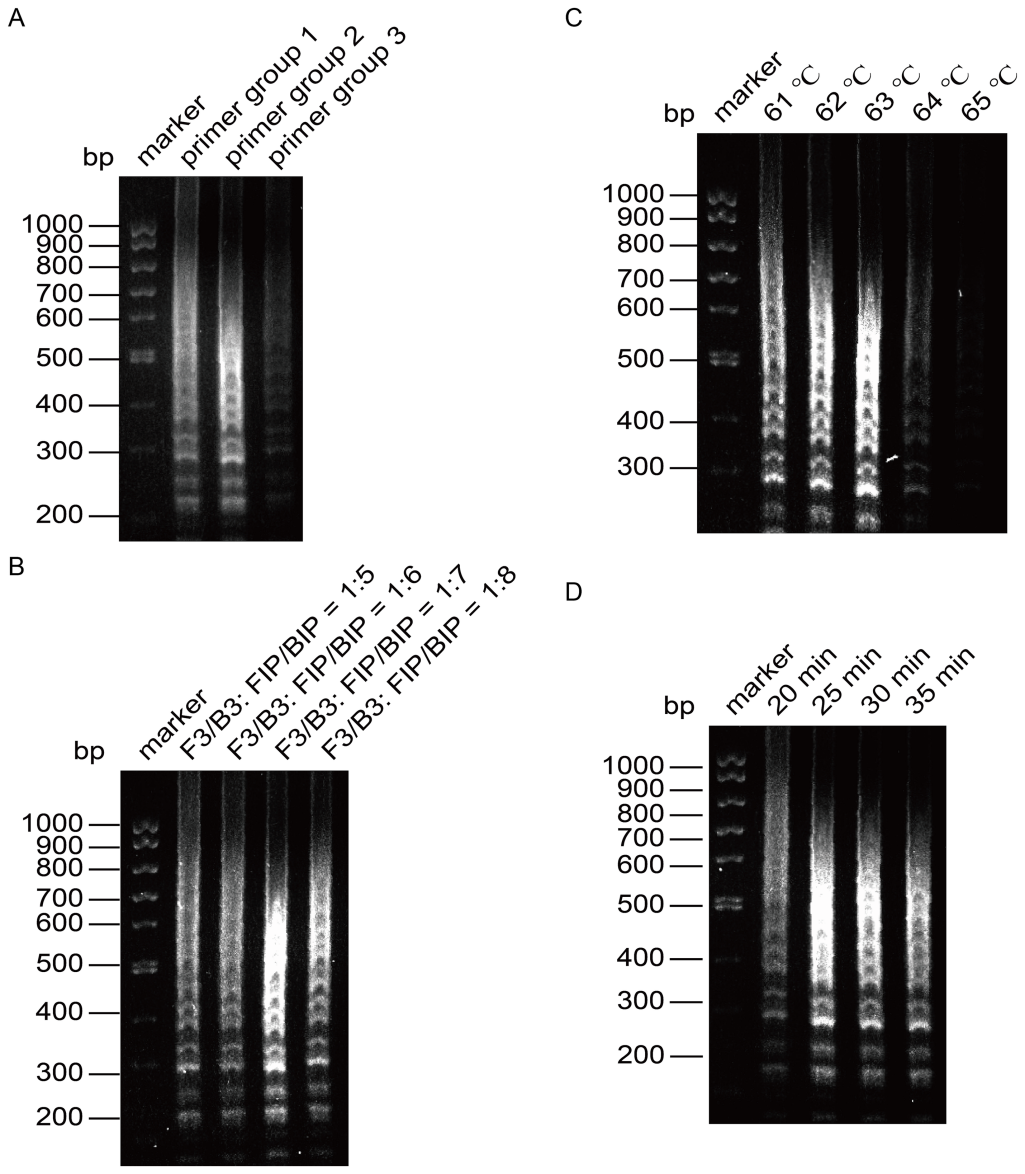
Optimization of the reaction system for LAMP detection of CHIKV. **(A)** Screening of primer groups. **(B)** Optimization of outer primer and inner primer ratios. **(C)** Optimization of reaction temperature. **(D)** Optimization of reaction time.

We optimized the reaction conditions for the primers targeting the CHIKV based on the established initial LAMP amplification system. The nucleic acid electrophoresis results shown in Figure 3A indicated that the three designed primer groups successfully conducted the amplification reaction, producing the characteristic ladder-like band pattern of positive amplification. However, primer groups 1 and 3 exhibited lighter bands compared to primer group 2, which displayed brighter and more pronounced positive bands with a more distinct step shape. Given that the positive bands of primer group 2 were brighter and had a ladder-like appearance, we selected primer group 2 as the optimal choice for CHIKV detection.

Next, we optimized the amplification temperature using primer group 2, setting six different ratios of outer to inner primers: 1:5, 1:6, 1:7, and 1:8. The amplified products were analyzed through agarose gel electrophoresis. As presented in Figure 3B, the amplification bands were brightest at a ratio of 1:7, leading us to determine that the optimal ratio of outer primer to inner primer for the reaction was 1:7.

With this optimal ratio established for primer group 1, we fixed the other reaction conditions and set six temperature gradients at 61°C, 62°C, 63°C, 64°C, and 65°C. The results, analyzed using agarose gel electrophoresis, indicated deeper bands when the reaction temperature was between 61°C and 63°C, with the most pronounced band gradient observed at 63°C (Figure 3C). Therefore, 63°C was selected as the optimal reaction temperature.

Once the optimal primers and reaction temperature were fixed, we evaluated four time gradients: 20 min, 25 min, 30 min, and 35 min. After analyzing the amplified products via agarose gel electrophoresis, we found that the bands were faint at 20 min, but peaked at 25 min, showing clear intensity (Figure 3D). To enhance reaction efficiency, we determined 25 min as the optimal amplification time, with the most distinct bands observed at 63°C.

**Figure 4:**
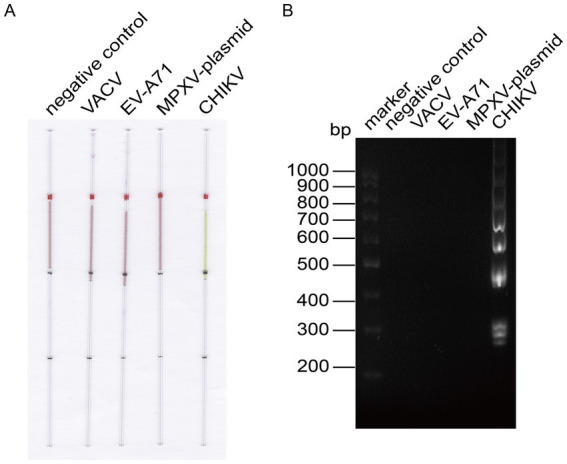
LAMP reaction system for detecting CHIKV specificity. **(A)** Capillary-modified LAMP detection of CHIKV specificity. From left to right: negative control, 100 ng VACV DNA, 100 ng EV-A71 RNA, 100 ng MPXV F13 plasmid, 100 ng CHIKV RNA. This capillary reaction system utilized the HotStart Bst 4.0 LHNB Lyo Mix that features a violet (negative) to green (positive) color change. **(B)** Validation of CHIKV-specific products through agarose gel electrophoresis.

To verify the specificity of the established LAMP reaction system, we employed the capillary modified LAMP assay to detect CHIKV RNA along with other virus RNA or plasmids available in the laboratory, such as Enterovirus A71 (EV-A71) RNA, VACV DNA, and MPXV F13 plasmid. The amplified products were analyzed through agarose gel electrophoresis to validate the specificity of the established LAMP method, as illustrated in Figure 4. The specificity analysis showed a distinct color change in the CHIKV group, while no such color change was observed in the other groups (Figure 4A). Electrophoresis results (Figure 4B) revealed prominent bands in the CHIKV group, while no bands were present in the other groups, indicating that the specificity of the system was robust.

**Figure 5:**
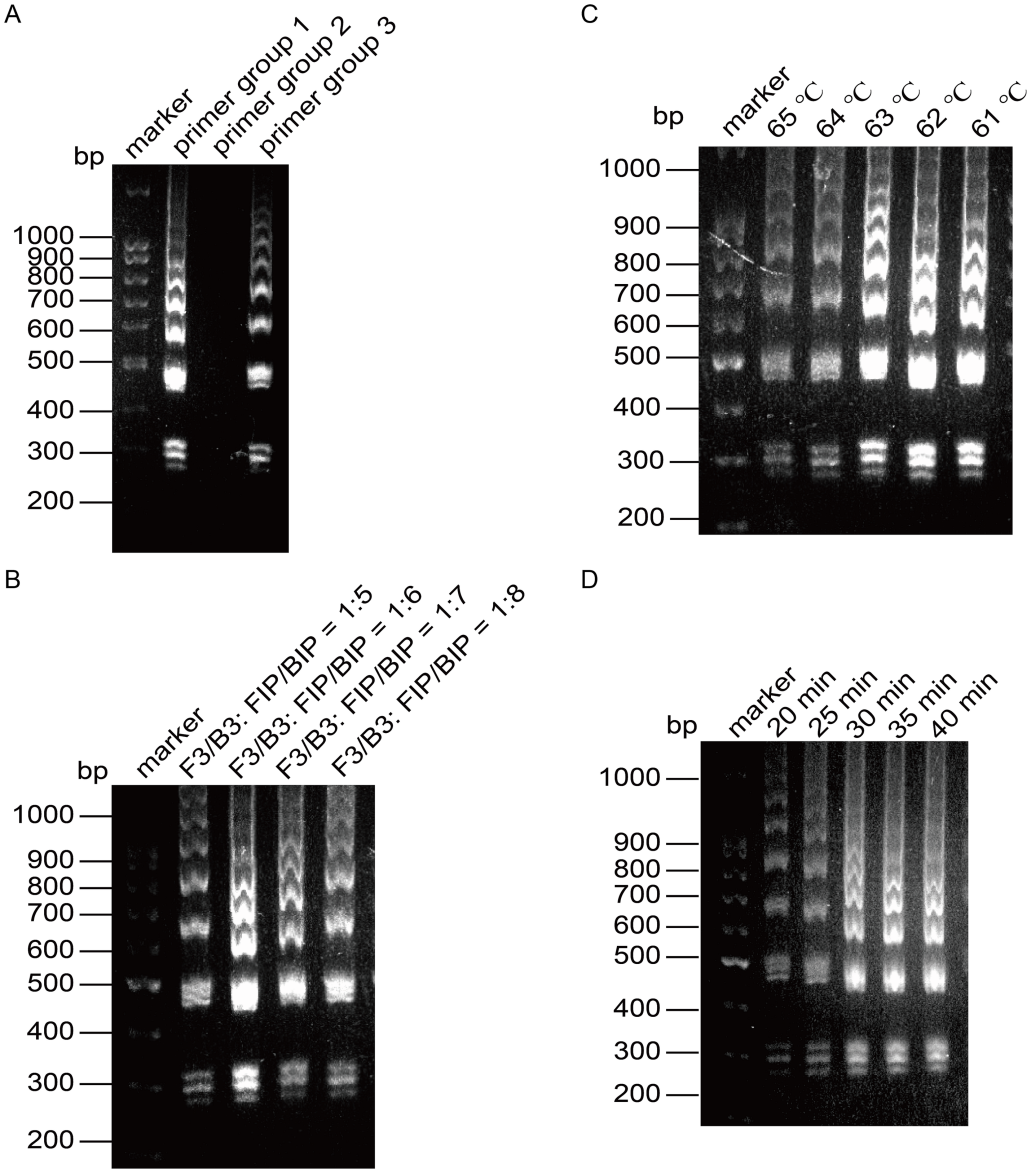
Optimization of the reaction system for LAMP detection of VACV. **(A)** Screening of primer groups. **(B)** Optimization of outer primer and inner primer ratios. **(C)** Optimization of reaction temperature. **(D)** Optimization of reaction time.

**Figure 6:**
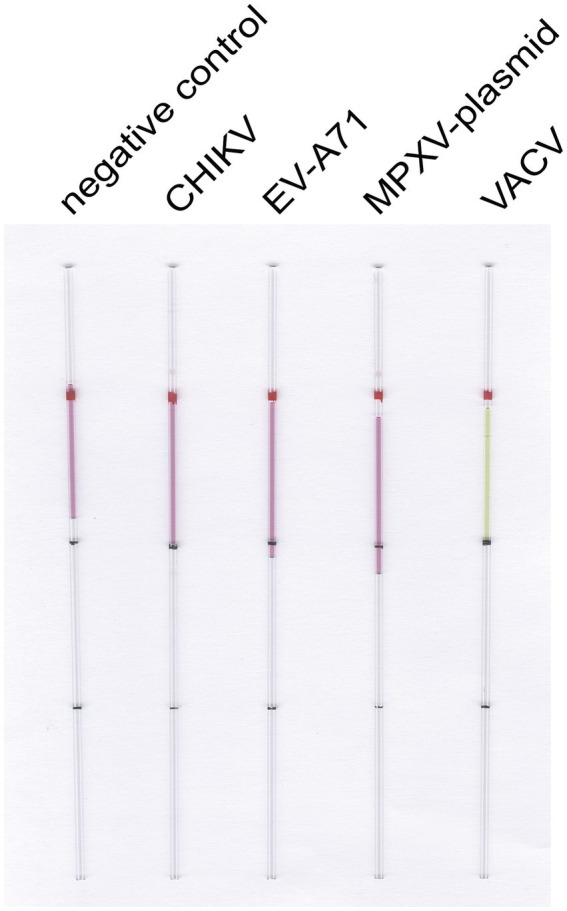
LAMP reaction system for detecting VACV specificity. Capillary-modified LAMP detection of VACV specificity. From left to right: negative control, 100 ng CHIKV RNA, 100 ng EV-A71 RNA, 100 ng MPXV F13 plasmid, 100 ng VACV DNA. This capillary reaction system utilized the HotStart Bst 4.0 LHNB Lyo Mix that features a violet (negative) to green (positive) color change.

Similarly, we performed the same optimization for VACV (Figure 5), determining the optimal primer set, the optimal ratio of F3/B3 to FIP/BIP as 1:6, the optimal reaction temperature at 63°C, and the optimal reaction time at 30 min. We then employed the capillary modified LAMP assay to detect VACV in conjunction with other viruses and plasmids available in our laboratory, including EV-A71, CHIKV, and the MPXV plasmid. The results demonstrated that our assay is specific for VACV (Figure 6).

### 3.3 The capillary-modified LAMP detection of MPXV F13 gene fragment in a plasmid

**Figure 7:**
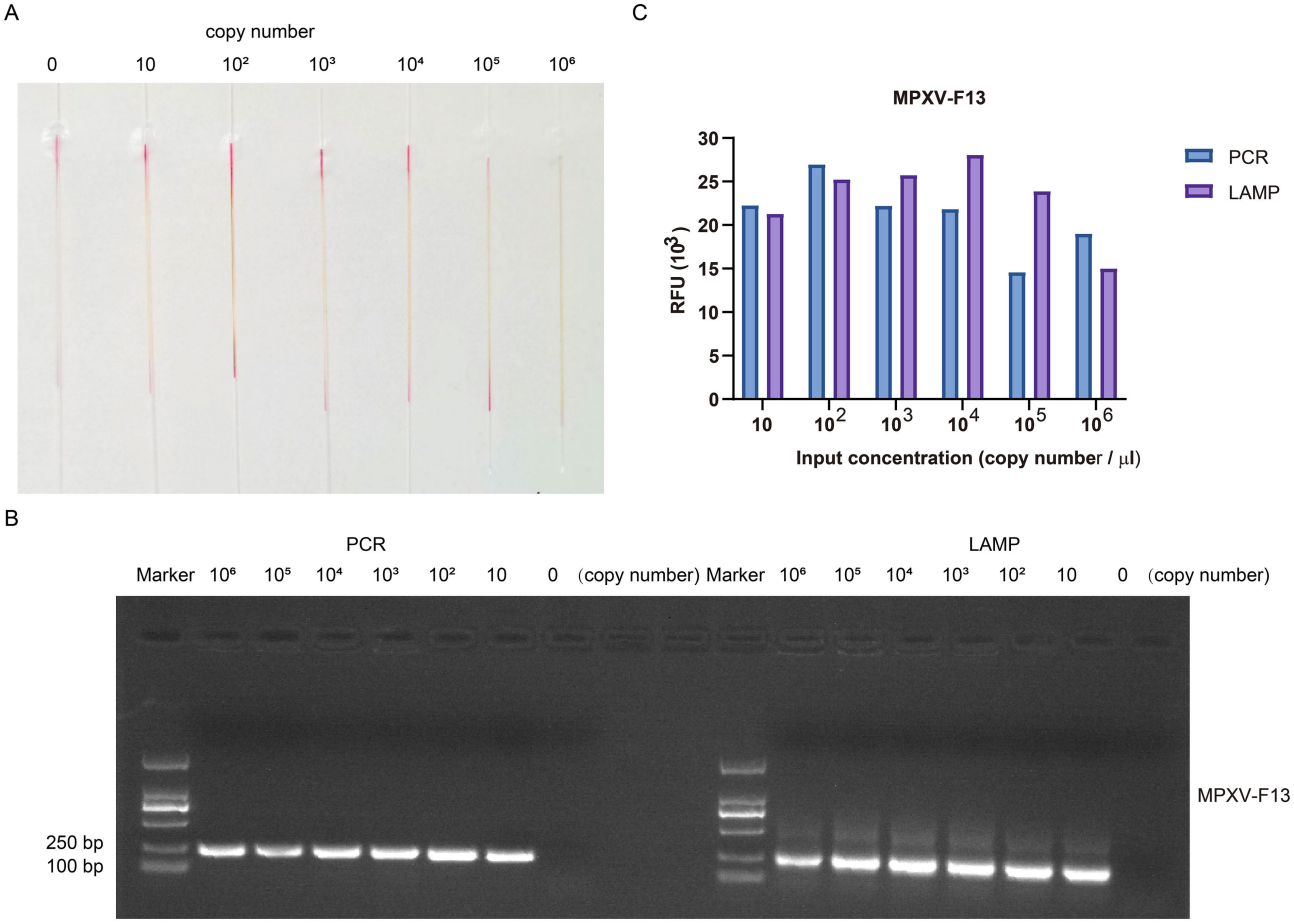
The gradient detection experiments of the plasmid containing MPXV F13 gene insert by capillary-modified LAMP amplification. **(A)** The plasmids containing MPXV F13 gene insert were serially diluted from 10 copies/μl to 1 × 10^5^ copies/μL. This capillary reaction system utilized the Bst 4.0 LS MasterMix that features a red (negative) and yellow (positive) color change. **(B)** Comparison of PCR and LAMP amplification ability using agarose gel electrophoresis analysis. **(C)** Comparison of detection signals of PCR and LAMP amplification.

To assess the sensitivity of our method, we conducted gradient detection experiments using a 10-fold dilution series of the plasmid containing the MPXV F13 gene insert. For the control group, double distilled water was used instead of the plasmid containing viral gene inserts. The concentration of the plasmid containing viral gene insert was determined using a spectrophotometer nanodrop8000. By applying the formula for calculating copy numbers, the DNA copy number of the constructed MPXV F13 plasmid was estimated to be approximately 1.0 × 10^11^ copies/μL. The plasmid was then diluted 10-fold to reach the standard concentration. Microcapillary NO. 1 served as the control group, while microcapillaries NO. 2 to NO. 7 contained copy number concentrations of 10 copies/μL, 1 × 10 copies/μL, 1 × 10^2^ copies/μL, 1 × 10^3^ copies/μL, 1 × 10^4^ copies/μL, and 1 × 10^5^ copies/μL, respectively (Figure 7A). The results showed that the reaction system in the microcapillaries changed color from red to yellow at a copy number concentration of 10 copies/μL, indicating the high sensitivity of this method. We used PCR to target the same genes as LAMP (Figure 7B). As a result, we found that the sensitivity of LAMP was comparable to PCR. In the MPXV F13 plasmid amplification assay, LAMP provided a stronger detection signal (Figure 7C), and the results demonstrated the improved sensitivity of our capillary-modified LAMP assay compared to PCR.

### 3.4 The capillary-modified LAMP detection of VACV

**Figure 8:**
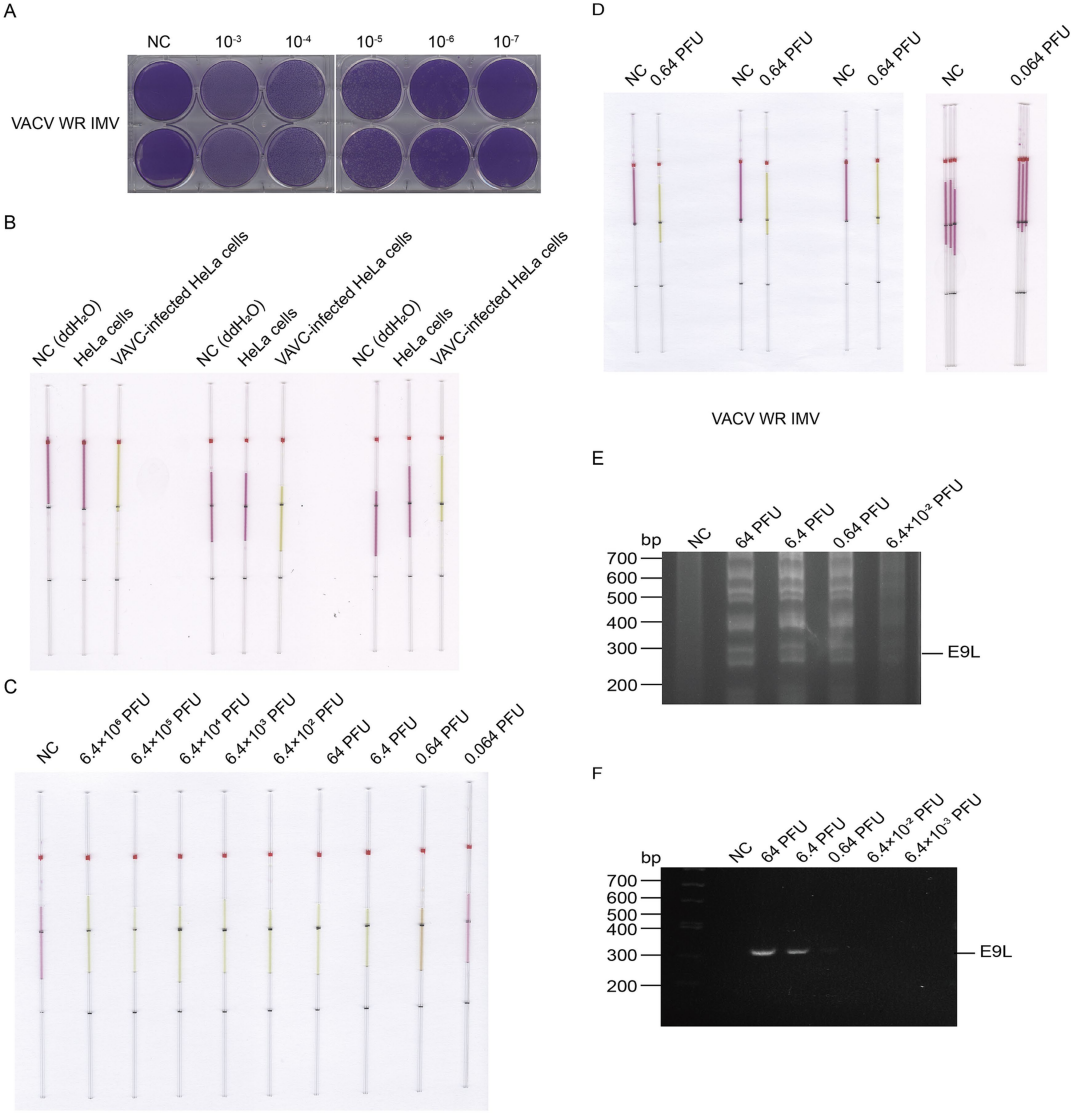
Detection of VACV cell lysate DNA by capillary-modified LAMP amplification and minimum detection limit corresponding to viral plaque forming unit. **(A)** Determination of viral titers in VACV cell lysate. **(B)** Comparison of cell lysates from VACV-infected and uninfected control cells. Experiments were conducted in triplicate. This capillary reaction system utilized the HotStart Bst 4.0 LHNB Lyo Mix that features a violet (negative) to green (positive) color change. **(C)** Sensitivity of staining-based LAMP color change reaction for DNA detection, the DNA fragments were extracted from viral cell lysate ranged from 0.064 to 6.4 × 10^6^ PFU, ddH_2_O is used as a negative control. This capillary reaction system utilized the HotStart Bst 4.0 LHNB Lyo Mix that features a violet (negative) to green (positive) color change. **(D)** Repeated assessment of the detection and undetectable limit for VACV IMV. This capillary reaction system utilized the HotStart Bst 4.0 LHNB Lyo Mix that features a violet (negative) to green (positive) color change. **(E)** Validation of VACV WR strain cell lysate DNA amplification products by agarose gel electrophoresis. **(F)** Validation of PCR amplification products using agarose gel electrophoresis.

Subsequently, We collected the cell lysate after virus expansion, diluted the cell lysate serial and performed plaque experiments, and measured the plaque formation unit of 4 × 10^5^ PFU/mL (Figure 8A). To verify the specificity of the designed primers and to exclude any non-specific amplification of host background nucleic acids, we established a series of control experiments. These included virus-positive samples, host-negative samples, and ddH_2_O control, with each experiment being repeated three times. The results demonstrated that the primers successfully amplified the expected product in the virus-positive samples, accompanied by a noticeable color change. In contrast, no color change was observed in the host background nucleic acid group or the ddH_2_O control (Figure 8B), indicating that no non-specific amplification reactions occurred. The viral cell lysate was gradually diluted so that the viral dosage in the cell lysate was 6.4 × 10^6^ PFU, 6.4 × 10^5^ PFU, 6.4 × 10^4^ PFU, 6.4 × 10^3^ PFU, 6.4 × 10^2^ PFU, 64 PFU, 6.4 PFU, 0.64 PFU and 0.064 PFU, respectively. We extracted DNA fragments from viral cell lysate ranged from 0.064 to 6.4 × 10^6^ PFU to assess the ability of our capillary-modified LAMP to detect VACV. As shown in Figure 8C, the detection limit for VACV viral cell lysate was 0.64 PFU. All experiments were conducted using a strict repeated design, with each sample independently tested three times. We assessed the lower detection limits of infectious units for VACV IMV, repeating the evaluation for undetectable units. The experimental data are presented in Figure 8D. The DNA amplification products of the VACV cell lysate were verified by agarose gel electrophoresis (Figure 8E), and the results were consistent. We targeted the same E9L gene for the PCR reaction. The results indicated that the detection limit for VACV viral cell lysate was 6.4 PFU (Figure 8F). This suggests that the sensitivity of our capillary modified LAMP assay is 10 times higher than that of PCR.

**Figure 9:**
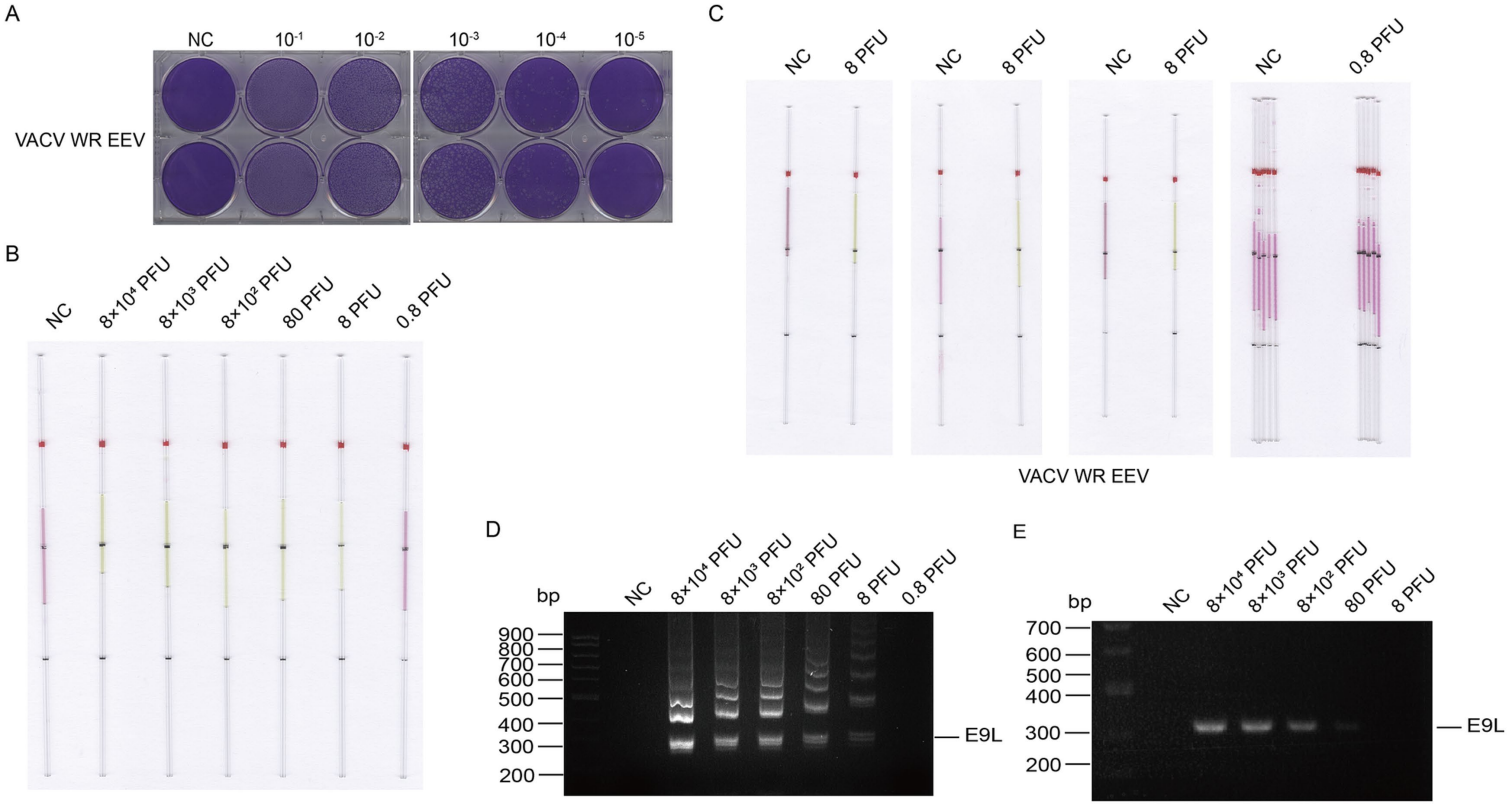
Detection of VACV supernatant DNA by capillary-modified LAMP amplification and minimum detection limit corresponding to viral plaque forming unit. **(A)** Determination of viral titers in VACV supernatant. **(B)** Sensitivity of staining-based LAMP color change reaction for DNA detection, the DNA fragments were extracted from supernatant ranged from 0.8 to 8 × 10^4^ PFU, ddH_2_O is used as a negative control. This capillary reaction system utilized the HotStart Bst 4.0 LHNB Lyo Mix that features a violet (negative) to green (positive) color change. **(C)** The repeated experiments to determine the detection and undetectable limits of infectious units for VACV EEV. This capillary reaction system utilized the HotStart Bst 4.0 LHNB Lyo Mix that features a violet (negative) to green (positive) color change. **(D)** Validation of DNA amplification products from the VACV WR strain supernatant using agarose gel electrophoresis. **(E)** Validation of PCR amplification products through agarose gel electrophoresis.

Similarly, the supernatant of VACV was tested for plaque formation and sensitivity. DNA fragments extracted from the supernatant ranged from 0.8 PFU to 8 × 10^4^ PFU (Figures 9A,B). We conducted three repeated experiments to determine the detection limits of infectious units for VACV EEV, and repeated the assessment five times for undetectable units (Figure 9C). The LAMP detection limit for VACV supernatant was 8 PFU (Figure 9D), while the detection limit for PCR was 80 PFU (Figure 9E), demonstrating that LAMP is indeed 10 times more sensitive than PCR.

### 3.5 The capillary-modified LAMP detection of CHIKV nsP1 gene fragment in a plasmid

**Figure 10:**
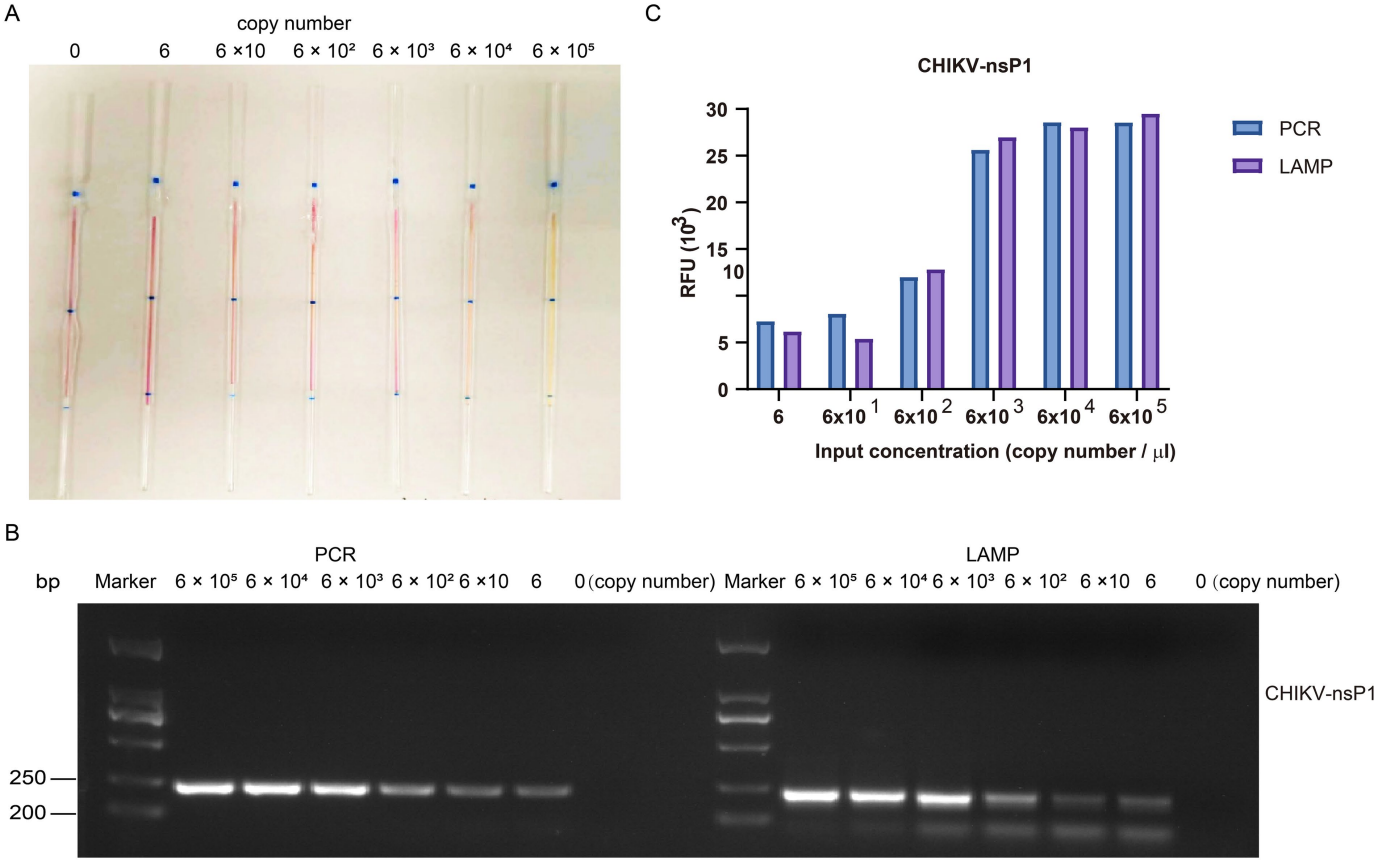
The gradient detection experiments of the plasmid containing CHIKV nsP1 gene insert by capillary-modified LAMP amplification. **(A)** The plasmids containing CHIKV nsP1 gene insert were serially diluted from 6 copies/μl to 6 × 10^5^ copies/μL. This capillary reaction system utilized the Bst 4.0 LS MasterMix that features a red (negative) and yellow (positive) color change. **(B)** Comparison of PCR and LAMP amplification ability using agarose gel electrophoresis analysis. **(C)** Comparison of detection signals of PCR and LAMP amplification.

Similarly, the DNA copy number of the constructed CHIKV nsP1 plasmid was calculated to be approximately 6.5 × 10^10^ copies/μL. The plasmid was diluted tenfold to reach the standard concentration of 6 copies/μL, 6 × 10 copies/μL, 6 × 10^2^ copies/μL, 6 × 10^3^ copies/μL, 6 × 10^4^ copies/μL, and 6 × 10^5^ copies/μL, respectively (Figure 10A). The results showed that the reaction system in the microcapillaries changed color from red to yellow at a concentration of 6 × 10 copies/μL. We also used PCR to target the same genes as LAMP (Figure 10B) and found that the sensitivity of LAMP was comparable to PCR (Figure 10C). The results demonstrate the high sensitivity of our capillary modified LAMP assay.

### 3.6 The capillary-modified LAMP detection of CHIKV live virus

**Figure 11:**
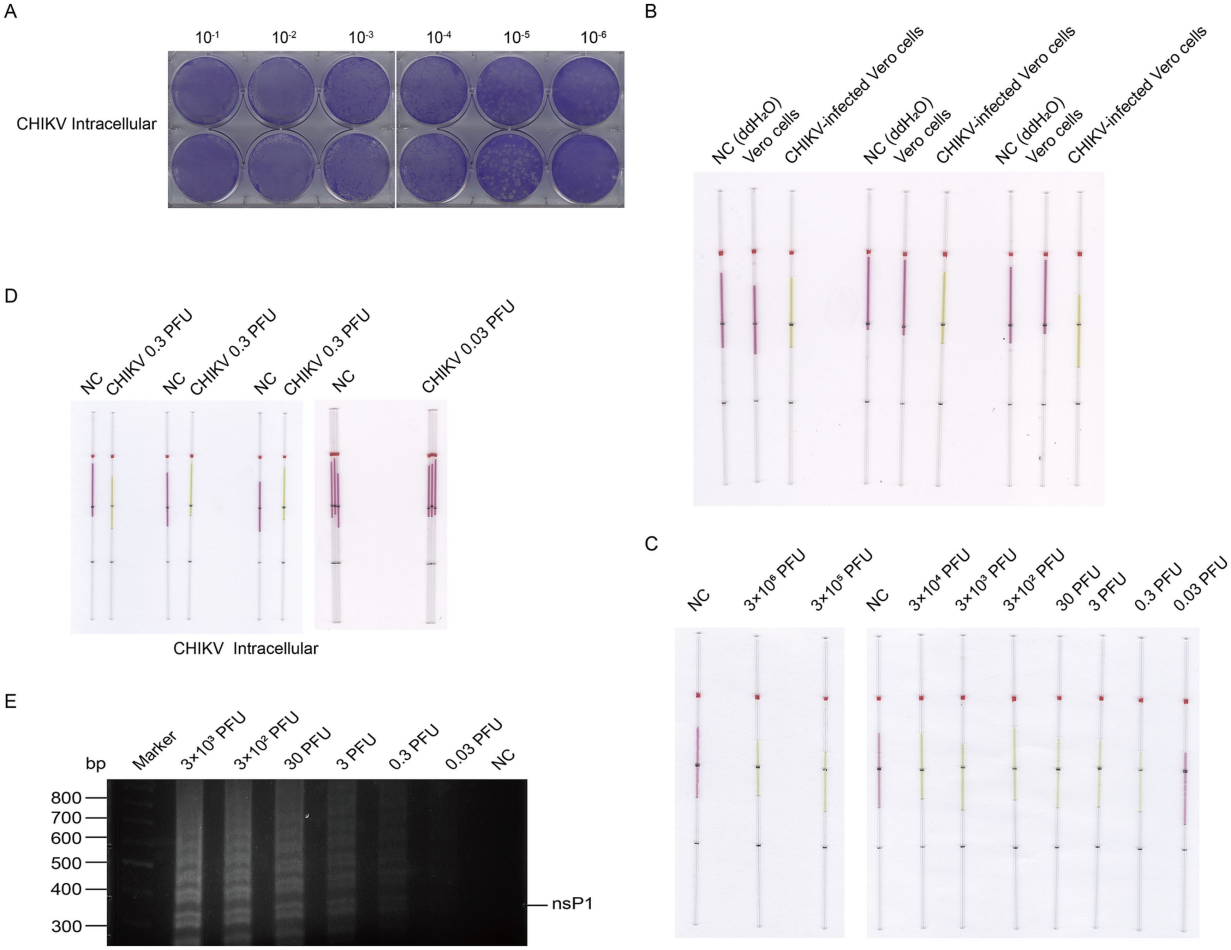
Detection of CHIKV from cell lysate RNA by capillary-modified LAMP amplification and minimum detection limit corresponding to viral plaque forming unit. **(A)** Determination of viral titers in CHIKV cell lysate. **(B)** Comparison of cell lysates from CHIKV-infected and uninfected control cells. Experiments were conducted in triplicate. This capillary reaction system utilized the HotStart Bst 4.0 LHNB Lyo Mix that features a violet (negative) to green (positive) color change. **(C)** Sensitivity of staining-based LAMP color change reaction for RNA detection, the RNA fragments were extracted from viral cell lysate ranged from 0.03 to 3 × 10^6^ PFU, ddH_2_O is used as a negative control. This capillary reaction system utilized the HotStart Bst 4.0 LHNB Lyo Mix that features a violet (negative) to green (positive) color change. **(D)** Repeated assessment of the detection and undetectable limit for CHIKV from cell lysate. This capillary reaction system utilized the HotStart Bst 4.0 LHNB Lyo Mix that features a violet (negative) to green (positive) color change. **(E)** Validation of CHIKV cell lysate RNA amplification products by agarose gel electrophoresis.

**Figure 12:**
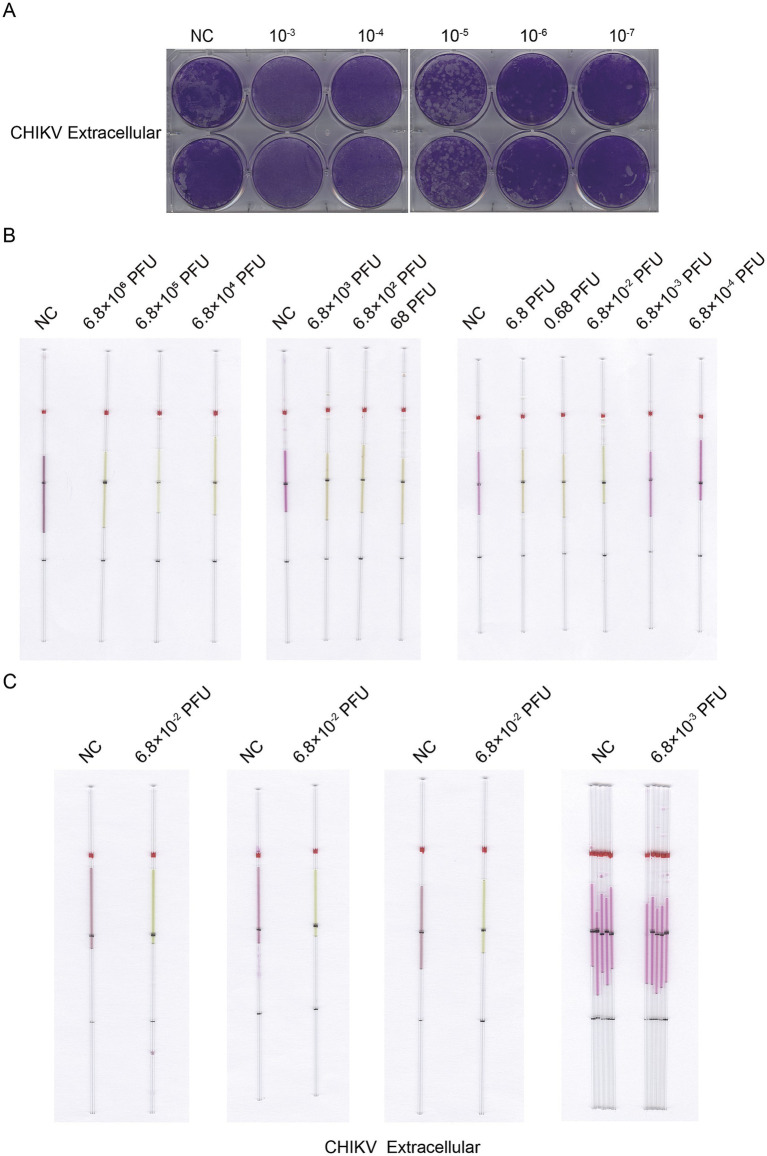
Detection of CHIKV from supernatant RNA by capillary-modified LAMP amplification and minimum detection limit corresponding to viral plaque forming unit. **(A)** Determination of viral titers in CHIKV supernatant. **(B)** Sensitivity of staining-based LAMP color change reaction for RNA detection, the RNA fragments were extracted from supernatant ranged from 6.8 × 10^−4^ to 6.8 × 10^6^ PFU, ddH_2_O is used as a negative control. This capillary reaction system utilized the HotStart Bst 4.0 LHNB Lyo Mix that features a violet (negative) to green (positive) color change. **(C)** The repeated experiments to determine the detection and undetectable limits of infectious units for CHIKV from supernatant. This capillary reaction system utilized the HotStart Bst 4.0 LHNB Lyo Mix that features a violet (negative) to green (positive) color change.

We used the same method to extract and detect the viral CHIKV RNA as the detection of VACV. We extracted RNA from the lysates of cells infected with CHIKV at doses ranging from 0.03 to 3 × 10^6^ PFU. We determined a lower limit of detection of 0.3 PFU for the cell lysate of CHIKV (Figure 11) and 0.068 PFU for the supernatant (Figure 12).

## 4 Discussion

MPXV and CHIKV have received international attention in recent years as viruses that could cause major epidemics. Mpox exhibits symptoms similar to smallpox and can lead to severe complications including death (Gupta et al., 2023). Moreover, the virus has been evolving and showing atypical clinical manifestations which posed a significant risk of covert transmission (Palmore and Henderson, 2022). Since the outbreak began in May 2022, the disease has been reported in regions where mpox transmission had not been documented previously (Kiely et al., 2023; Xu and Zhang, 2023). The number of cases and the affected areas grew rapidly, indicating a rapid shift in the trend of MPXV spread from endemic to non-endemic countries. Non-endemic countries now face a higher risk of imported cases. CHIKV is mainly transmitted through the bites of *Aedes aegypti* and *Aedes albopictus* mosquitoes, which are widely distributed and can easily cause large-scale outbreaks (Zeller et al., 2016). The transmission route, epidemiological characteristics, and clinical symptoms of CHIKV are very similar to those of dengue virus. Due to the covert and contagious nature of these two viruses, accuracy and efficiency are crucial during entry quarantine. Port inspection agencies and primary health care providers urgently need a new testing method that is inexpensive, simple, rapid, relatively accurate, and does not require specialized laboratory equipment.

Current PCR-based diagnostics, while analytically robust, face implementation barriers in resource-limited settings. In contrast, LAMP offers distinct advantages including rapid turnaround, minimal equipment requirements, and operational simplicity (Nam et al., 2023). The Bst 4.0 enzyme we use is capable of performing reverse transcription LAMP (RT-LAMP), which integrates both reverse transcription and DNA amplification into a single-enzyme system, eliminating the need for additional reverse transcriptase. This streamlined approach enhances amplification efficiency and adaptability, facilitating the detection of RNA viruses similar to CHIKV. In contrast, PCR detection of RNA viruses necessitates a separate reverse transcription step, during which RNA degradation may occur, thereby reducing the initial template available for cDNA synthesis. Additionally, the efficiency of reverse transcriptase is not 100%, meaning that some RNA molecules may not be fully reverse transcribed, which can impact the final concentration of cDNA. Consequently, we primarily focus on the detection of DNA viruses (VACV) to evaluate the advantages of LAMP amplification sensitivity. Building on these strengths of LAMP detection, our study implemented three critical modifications to the standard LAMP protocols: (1) using capillary tubes as reaction chambers; (2) Optimization of reaction system to accommodate reduced reaction volumes; (3) Incorporating of a LHNB dye in the detection system for visual readout compatibility.

The substitution of conventional centrifuge tubes with capillary as reaction vessels addressed multiple technical limitations. Capillary tubes are more suitable for reactions with decreased volume of reagents and samples. When we first attempted to reduce the volume of reagents and samples, inadequate reaction and reagent wastage occurred due to adhesion to the wall of centrifuge tube. By switching to capillary tubes, we solved this problem. The optimized MPXV amplification system still achieved good results in capillary tubes even when the volume was reduced to 10 μL, reducing the amount of reagents used per test, thus further lowering costs. In clinical scenarios involving rare samples, a 10 μL system can minimize template consumption and reduce the cost of each detection, making it practically significant for studies with limited sample sizes. The capillary’s capillary action also eliminates the need for relatively expensive pipettes, making it possible to produce and distribute simple detection kits with high sensitivity to general health care providers on a massive scale, giving this method the potential to achieve point-of-care testing (POCT). Capillary tubes is better when it comes to heat distribution, which helps control the temperature stability and consistency of the LAMP amplification system. Last but not least, the color change in capillary tubes is more apparent, facilitating visual determination of the test results.

Theoretically, due to the specificity of the outer primer, inner primer, and loop primer, as well as the efficient strand displacement property of Bst polymerase, LAMP offers high sensitivity. We validated the sensitivity of our modified method with the gradient detection experiments of the plasmid containing viral gene insert. For further demonstrating the strong amplification activity of the capillary-modified LAMP reaction system while reducing reagent usage, we conducted gel electrophoresis experiments to detect CHIKV nsP1 plasmids and MPXV F13 plasmids amplified using both PCR and LAMP techniques. The results indicated that the optimized LAMP reaction system had higher amplification activity compared to PCR. Thus we think the slightly lower sensitivity observed in the gradient detection experiments of the plasmid containing viral gene insert compared to qPCR detection may be due to the lower sensitivity of visual inspection compared to the fluorescence signal of qPCR, which could result in a slight lower analytical sensitivity for visual LAMP assays. Nevertheless, our method still offers high sensitivity that can fully meet practical application requirements.

Subsequently, for the CHIKV, we conducted tests on its live virus, and the high sensitivity of this detection method was validated. Regarding MPXV detection, due to the highly biosafety level required for MPXV cultivation (BSL-3) (Hughes et al., 2017), we used VACV as an ideal substitute for testing *Orthopoxviruses* because of its high similarity to other poxviruses. The selected E9L gene can target all poxviruses (Zhao et al., 2023). Similarly, the detection method also demonstrated high sensitivity for VACV. This method can lay a solid foundation for early detection and prevention of CHIKV and MPXV infections.

Moreover, in our research, we found that the sensitivity of detecting nucleic acids extracted from lysates of VACV-infected cells was an order of magnitude higher than that from the supernatant. In contrast, the results for detecting CHIKV were the opposite. This discrepancy is due to the generation of a large amount of non-infectious forms during the complex morphogenesis process of VACV (Condit et al., 2006). In contrast, CHIKV does not produce as many non-infectious particles within infected cells (Solignat et al., 2009), leading to the contrary outcome.

The capillary-modified visual LAMP testing method for MPXV and CHIKV developed in this study exhibited robust consistency and sensitivity. Compared to qPCR, our method demonstrates superior operational practicality for field deployment through the following features: (1) simplified workflow requiring only a single-step reaction with capillary-mediated liquid handling; (2) specialized personnel independence through colorimetric interpretation (no specialized training or data analysis needed); (3) minimal instrumentation (compatible with standard heating blocks or water baths); (4) rapid turnaround time (30-min amplification cycle) and (5) cost efficiency (further reducing reagent usage compared to traditional LAMP). These attributes collectively enhance its suitability for point-of-care applications in resource-limited settings.

At this stage, our study does not encompass clinical samples, such as patient serum and skin lesion swabs, due to the stringent ethical reviews and informed consent processes required for their collection. However, we anticipate future collaboration with clinical organizations possessing such samples to conduct clinical trials that adhere to laboratory biosafety standards. This collaboration will allow us to validate the sensitivity and specificity of our method in real-world scenarios and develop supporting sample pre-treatment kits to enhance compatibility with complex samples. Implementing this optimized testing method could empower frontline healthcare workers with a rapid, equipment-minimized, and cost-effective diagnostic tool. This would strengthen early outbreak containment capabilities for MPXV and CHIKV in regions with limited laboratory infrastructure.

Visual detection sensitivity limits can significantly impact the effectiveness of LAMP assays in field applications. Low sensitivity in visual detection may lead to false-negative results, particularly in scenarios with low copy numbers. In field settings where resources are limited and confirmatory testing may not be available, these false negatives can result in undetected infections or pathogens, posing serious implications for public health and disease management. Moreover, the reliance on subjective visual interpretation can introduce variability. Environmental factors such as lighting conditions and the physical well-being of the observer can affect detection, resulting in inconsistent outcomes. This variability can undermine confidence in the test, especially in high-stakes situations. Additionally, in remote locations where the LAMP assay is intended to be utilized, limitations in visual sensitivity can hinder its adaptability, as practitioners may struggle to assess subtle color changes under varying environmental conditions. To enhance the reliability of LAMP assays in field settings and minimize false negatives, several strategies can be employed. Careful optimization of amplification conditions, such as magnesium ion concentration, temperature, and time, can improve the efficiency of the reaction and enhance visualization. Including positive and negative controls in each assay can help validate results, while internal control mechanisms can verify the integrity of the reaction and indicate proper amplification. Furthermore, utilizing more sensitive or dual-labeled dyes that produce a clear signal change upon amplification can enhance visual detection. Dyes that yield stronger, more distinct color changes would facilitate the identification of positive results. Providing standardized training for operators can help reduce variability in interpretation. Clear guidelines on visual detection thresholds and documentation can lead to more consistent assessments across various field settings. By implementing these strategies, the reliability of LAMP assays in field applications can be significantly improved, thereby reducing the chances of false negatives and enhancing overall diagnostic accuracy.

## 5 Conclusion

In this study, we successfully established a capillary-modified visual LAMP method for the rapid detection of MPXV and CHIKV. This novel approach exhibits higher sensitivity than qPCR while addressing some of the limitations that have impeded large-scale qPCR screening. The key features of this new method can be summarized as simplicity, rapidity, and cost-effectiveness, making high-sensitivity detection of MPXV and CHIKV accessible to primary health services and border quarantine facilities where it is needed the most. This advancement enables large-scale screening for MPXV and CHIKV during early epidemic windows, which is crucial for outbreak response, as it facilitates timely intervention and control measures. By bridging the diagnostic accessibility gap, this method adds a powerful tool to primary healthcare providers’ arsenal and significantly enhances epidemic management capabilities in low-income regions.

## Data Availability

The raw data supporting the conclusions of this article will be made available by the authors, without undue reservation.

## Footnotes

^1^ https://primerexplorer.eiken.co.jp/lampv5e/index.html

^2^ https://www.atcc.org/products/ccl-26

^3^ http://www.haigene.cn/html/show-73.html
